# Targeting ferroptosis and mitochondrial ROS: organoprotective mechanisms of anesthetic conditioning in liver transplantation

**DOI:** 10.3389/fmolb.2026.1901963

**Published:** 2026-08-19

**Authors:** Junming Li, Weijun Zeng, Juan Yang

**Affiliations:** The First Affiliated Hospital of Kunming Medical University, Kunming, Yunnan, China

**Keywords:** anesthetic conditioning, ferroptosis, graft protection, ischemia–reperfusion injury, lipid peroxidation, liver transplantation, mitochondrial reactive oxygen species, sevoflurane

## Abstract

Liver transplantation is the definitive treatment for advanced liver failure, yet ischemia-reperfusion injury (IRI) remains a major challenge—particularly for marginal, steatotic, or donation-after-circulatory-death (DCD) grafts. While oxidative stress and inflammation are long-recognized contributors to IRI, this review focuses on two tightly linked, mechanistically specific pathways: ferroptosis and mitochondrial reactive oxygen species (ROS). Together, these pathways convert reperfusion-associated metabolic stress into injury of hepatocytes, endothelium, and bile ducts. Anesthetic preconditioning has gained interest as a modulator of this injury cascade. Volatile anesthetics, alongside propofol and dexmedetomidine, show experimental efficacy in reducing IRI by stabilizing mitochondria, regulating redox status, preserving GPX4(Glutathione Peroxidase 4)/SLC7A11(Solute Carrier Family 7 Member 11)-mediated antioxidant capacity, limiting lipid peroxidation, and dampening innate immune responses. However, clinical translation is limited by heterogeneity in experimental models, variability in anesthetic regimens, overreliance on non-specific oxidative stress markers, and a lack of validated ferroptosis-related endpoints in human studies. In summary, ferroptosis and mitochondrial ROS provide a cohesive mechanistic framework for graft vulnerability at reperfusion. To advance this field, future clinical studies should move beyond general oxidative stress assessments toward biomarker-driven approaches—integrating ferroptosis-specific markers and mitochondrial function tests. Coupling these with risk stratification of donor livers based on biochemical profiles, and tracking clinically meaningful outcomes, will enable precision strategies to mitigate IRI. This review underscores the need for targeted mechanistic validation to translate anesthetic conditioning into effective clinical organ protection.

## Introduction

Ferroptosis is an increasingly recognized mechanism of liver graft injury due to the high metabolic rate of the liver, high content of iron and lipids, and sensitivity to redox changes occurring during ischemia-reperfusion (Yamada et al., 2020). Ferroptosis is defined by the occurrence of iron dependent lipid peroxidation, failure of the antioxidants’ protective mechanism, and the resultant oxidative degradation of cellular membranes (Latunde-Dada, 2017). The main elements involved in ferroptosis include the Glutathione Peroxide Pathway (GPX), System Xc-activity, availability of Glutathione (GSH), fatty acid remodeling mediated by ACSL4, ferritinophagy, and accumulation of labile iron (Chen et al., 2021). Cold storage and subsequent reperfusion may lead to mobilization of iron, mitochondrial stress, and phospholipid oxidation that create conditions favorable for ferroptotic injury (Kojima et al., 2024). However, caution needs to be taken when interpreting results regarding ferroptosis since many studies indicate ferroptosis based on a very limited number of marker panels without showing iron dependency, accumulation of lipid peroxides, pathway specific rescue and excluding other cell death mechanisms.

Mitochondrial reactive oxygen species provide another key mechanism in connecting ischemic metabolic arrest to reperfusion injury (Perrelli et al., 2011). Ischemia primes the mitochondria for injury through the depletion of ATP, calcium accumulation, dysfunction of the respiratory chain, and imbalance in metabolic substrates (Mihaylov, 2021). Upon restoration of oxygen, excess superoxide production and downstream oxidant generation may occur due to electron leakage from the respiratory chain upon sudden restoration of oxygen (Blokhina et al., 2003). The resulting oxidative burst may cause mitochondrial permeability transition, loss of membrane potential, mitochondrial DNA release, activation of the inflammasome and recruitment of inflammatory cells (Xu et al., 2025). Thus, mitochondrial ROS cannot be viewed as an indicator of a single nonspecific injury; low or localized levels of ROS may participate in adaptive signaling while excessive levels and/or uncontrolled mitochondrial ROS may contribute to lipid peroxidation, endothelial injury and hepatocellular death (Cruz and Kang, 2018). Thus, measuring only total ROS provides an incomplete view of the redox state of mitochondria.

Thus, ferroptosis and mitochondrial ROS are not independent processes. Mitochondrial ROS may enhance lipid peroxidation and antioxidant depletion while ferroptotic membrane injury may increase mitochondrial instability and inflammatory signaling (Su et al., 2019). These two mechanisms create a self-reinforcing cycle involving redox collapse, iron-dependent lipid damage, innate immunity activation and failure of sinusoidal microcirculation. The relationship among these mechanisms is particularly relevant in liver transplantation due to the abrupt onset of reperfusion under conditions of surgical stress, hemodynamic instability, inflammatory activation and varying graft quality (Liu and Man, 2023).

Anesthetic preconditioning has attracted interest as it may affect various parts of this injury cascade. Volatile anesthetics such as Sevoflurane and Isoflurane as well as intravenous drugs such as Propofol and Dexmedetomidine have been found to stabilize mitochondria in experimental models, decrease oxidative stress, improve antioxidant signaling, exhibit anti-inflammatory properties and attenuate ischemia reperfusion injury (Tan et al., 2024). Anesthetic preconditioning is also clinically applicable as it can be administered at multiple stages of the transplantation process (e.g., donor management, graft preservation, recipient anesthesia or early reperfusion) (Beck-Schimmer et al., 2008). However, the current evidence base for anesthetic preconditioning varies greatly. Experimental studies vary widely in terms of animal species used, ischemia models employed, anesthetic application times, durations of anesthetic exposure, doses administered and outcomes measured. Clinical studies are further complicated by variability in donor quality and health status, disease severity in recipients, surgical techniques employed, cold ischemia time, intraoperative hemodynamics management and immunosuppressive regimens.

Therefore, this review will evaluate if there is pathway-specific evidence that demonstrates anesthetic preconditioning can modify ferroptosis and mitochondrial ROS in liver transplantation. It will focus on pathway-specific evidence and methodological issues limiting translatability to the clinic along with recommendations for biomarkers for guiding organ protection in the context of liver transplantation.

Beyond mechanistic considerations, the clinical significance of mitigating ischemia-reperfusion injury is underscored by the substantial burden of postoperative complications. Liver transplantation is the second most common type of solid organ transplantation worldwide (Choudhury et al., 2024). Despite its life-saving potential, the post-transplant course is frequently complicated by a spectrum of adverse events. Biliary complications, encompassing strictures and leaks, occur in 5%–32% of recipients and represent a major source of morbidity (Fasullo et al., 2022). Vascular complications, including hepatic artery thrombosis and stenosis, affect 2%–25% of recipients and constitute the most frequent and severe vascular events following transplantation. Acute T cell-mediated rejection affects approximately 10%–30% of patients, while infections occur in up to 34% of recipients and are associated with significantly higher odds of in-hospital mortality and sepsis. Many of these complications, particularly biliary and vascular injury, are directly exacerbated by the ischemia-reperfusion insult to the graft, highlighting the critical need for interventions that protect against this initial injury cascade. The need to expand the donor pool by utilizing marginal grafts—including steatotic, elderly, and donation-after-circulatory-death livers—further amplifies the vulnerability to such complications, as these grafts possess diminished metabolic reserve and heightened susceptibility to oxidative and ferroptotic injury. Therefore, understanding the mechanistic pathways that drive graft injury is not merely an academic exercise, but a prerequisite for developing perioperative strategies that can meaningfully improve patient outcomes.

## Hepatic ischemia–reperfusion injury in liver transplantation

Although the liver’s response to hepatic ischemia–reperfusion (IR) injury is a well established determinant of postoperative function in liver transplant recipients, ischemia reperfusion injury itself is a layered injury process (Dar et al., 2019). The injury begins during the unstable period of the donor liver and continues throughout cold and warm ischemia (Mihaylov, 2021). After reperfusion of the graft occurs, the full extent of the injury is realized. Upon reperfusion, abrupt changes occur in oxygen availability, temperature, perfusion pressure, substrate delivery for metabolism and inflammatory activation (Roy and Secomb, 2021). Such abrupt changes are particularly detrimental to grafts that have already been compromised due to decreased metabolic reserve; examples include steatotic grafts, elderly donor grafts, grafts obtained via donation after circulatory death, and grafts subjected to long periods of cold ischemia (Abbas et al., 2024). Consistently within current literature, researchers have established IR injury to liver transplants as being directly related to primary non-function, early allograft dysfunction, biliary injury, increased risk of acute rejection and ultimately decreased graft survival. However, the mechanisms behind these processes vary among different research studies.

Hepatocytes and nonparenchymal cells switch from aerobic respiration to anaerobic respiration during the ischemic phase in order to conserve energy due to lack of oxygen (Parente et al., 2023). As such, the ATP stores are depleted resulting in loss of Na+/K + -ATPase pump function leading to disrupted ion gradient, cellular edema and subsequent calcium overloading (Borović et al., 2016). During the ischemic phase, mitochondria lose the ability to generate an electrochemical gradient and therefore produce an abnormal amount of reactive oxygen species upon reperfusion (Perrelli et al., 2011). Furthermore, succinate produced during the ischemic phase serves as a readily available source of electrons for the mitochondrial electron transport system once oxygen is restored thereby facilitating the production of reactive oxygen species upon reperfusion (Pell et al., 2016). Although some researchers describe the ischemic phase as “biochemically inert,” the description is inaccurate as ischemia is not a passive event but sets up biochemical conditions that will affect the degree of injury caused by reperfusion (Kalogeris et al., 2017).

Upon initiation of reperfusion of the graft, there is a conversion of metabolic priming to oxidative and inflammatory injury. Restoration of oxygen at the onset of reperfusion allows for electron acceptors to be replenished but results in excessive production of superoxide radicals in mitochondria due to impaired electron transport (Chouchani et al., 2016). Oxidative damage subsequently occurs to proteins, lipids and DNA in mitochondria as well as plasma membranes. Simultaneously, calcium overload and opening of the mitochondrial permeability transition pore further impede oxidative phosphorylation and promote cell death signaling pathways (Morciano et al., 2021). In addition, reperfusion activates Kupffer cells, liver sinusoidal endothelial cells, neutrophils, platelets and complement pathways creating a vicious cycle where mitochondrial dysfunction produces sterile inflammation producing endothelial swelling promoting leukocyte adhesion creating microvascular hypoxia and reciprocally promoting each other (Kaltenmeier et al., 2022). This explains why restoring circulation does not lead to immediate recovery of tissue homeostasis; in fact, restoration of circulation may even exacerbate the injury initially caused by ischemia.

Currently, much of what exists in terms of literature regarding hepatic ischemia-reperfusion injury can be characterized as being overly simplistic in nature by describing it in general terms of oxidative stress and inflammation. While both descriptions are accurate in a very general sense, they are grossly inadequate in providing mechanisms of action at a high resolution level. As such, numerous experimental and clinical studies have utilized serum aminotransferases, histologic necrosis, malondialdehyde, total ROS, TNF-α levels, IL-6 levels and myeloperoxidase activity as common end points (Tsai et al., 2014). While these end points provide a general estimate of overall injury burden they do not allow investigators to determine whether mitochondrial-derived ROS was generated versus NADPH oxidase derived ROS, lipid peroxidation occurred vs. ferroptosis occurred, apoptosis occurred vs. necroptosis occurred or if sterile inflammatory necrosis occurred versus regulated cell death.

Thus, interventions may appear beneficial based on reduction in broad-based end point measures without clarity as to the actual molecular targets. This issue is critical in liver transplantation since multiple forms of cell death and stress pathways exist concurrently. For example, hepatocytes can undergo mitochondrial permeability transition mediated necrosis, apoptosis and ferroptosis depending on the magnitude and duration of injury (Zhang et al., 2021). Additionally, cholangiocytes can suffer from ischemic-cholangiopathy primarily due to sensitivity of the peribiliary vascular plexus to microcirculatory failure (Deltenre and Valla, 2006; Nakanuma and Miyata, 2017). Liver sinusoidal endothelial cells swell and activate prior to reperfusion through disruption of glycocalyx, imbalance of nitric oxide production and adhesion of leukocytes (Gracia-Sancho et al., 2021). Kupffer cells then amplify sterile-inflammation by releasing cytokines, chemokines and ROS (Huang et al., 2025). Finally, neutrophil and platelet adhesion occlude sinusoids and enhance endothelial injury (Wang and Liu, 2021). Therefore, the pathophysiology of hepatic ischemia-reperfusion injury must be viewed as a multi-cellular phenomenon and not simply an event occurring solely within hepatocytes.

Specific attention needs to be directed toward the biliary component of ischemia-reperfusion injury. Biliary complications after liver transplantation are not solely a result of surgical technique. Injury to cholangiocytes and the peribiliary microcirculation by ischemia leads to non-anastomotic stricture formation and ischemic cholangiopathy (Shi et al., 2023). Of note cholangiocytes have a more limited capacity for regeneration compared to hepatocytes after severe ischemic damage (Nwaduru et al., 2025). Moreover cholangiocytes require greater dependence on arterial microvascular supply compared to hepatocytes (Gaudio et al., 2006). Thus microvascular preservation and protection of endothelial integrity is paramount for graft survival (Parente et al., 2023). However many mechanistic studies underestimate biliary-related outcomes and focus almost exclusively on release of hepatocellular enzymes. Thus there exists a significant translational gap. An intervention that reduces early hepatocyte injury does not automatically preclude later biliary-related complications.

Furthermore the role of inflammation is more complex than previously thought as either good or bad. Kupffer cell activation can significantly exacerbate injury through release of TNF-a, IL-1b, IL-6, ROS and chemokines however macrophages are also necessary for removal of debris, modulation of immune responses and repair/tissue remodeling functions (Zimmermann et al., 2012). Similarly while neutrophil adherence to endothelium can cause significant damage to endothelial cells causing sinusoidal obstruction and other functional defects suppression of all innate immunity may compromise host defenses against infection and limit tissue regeneration (Konradt and Hunter, 2018). When designing protective strategies against organ injury it is essential that any intervention does not simply suppress inflammation indiscriminately but inhibits initial injurious inflammatory amplifications while maintaining reparative/inflammatory signals.

With increasing numbers of marginal donors being used for liver transplantation precise definition of mechanistic pathways involved in ischemia-reperfusion injury becomes increasingly urgent. Grafts that are steatotic contain excess amounts of lipid substrates that render them susceptible to lipid peroxidation and mitochondrial dysfunction (Shi et al., 2021). Donation after circulatory death grafts undergo unavoidable periods of warm ischemia prior to procurement making them uniquely susceptible to ATP depletion and resultant endothelial dysfunction and biliary ischemia (Mihaylov, 2021). Older donor livers have diminished mitochondrial tolerance or resistance to oxidative stress and reduced capacity for regeneration (Chatterjee et al., 2025). Longer durations of cold ischemia induce greater degrees of mitochondrial respiratory-chain dysfunction and endothelial activation (Villalba-López et al., 2023).

These characteristics make ischemia-reperfusion injury unique to transplantation context; thus mechanisms that are dominant in lean brain dead donor grafts may not be equally dominant in steatotic DCD grafts. Therefore detailed understanding of mechanisms involved in ischemia-reperfusion injury is crucial for development of effective therapeutic interventions aimed at minimizing graft injury/survival.

The following table summarizes the major cellular compartments involved in hepatic ischemia–reperfusion injury and links each compartment to dominant redox, inflammatory, and ferroptotic mechanisms are shown in Table 1.

**TABLE 1:** Cellular targets and mechanistic injury pathways in liver transplantation.

Cellular compartment	Dominant injury mechanism	Ferroptosis-relevant features	Mitochondrial ROS-Relevant features	Clinical relevance
Hepatocytes (Rampes and Ma, 2019)	ATP depletion, calcium overload, lipid membrane injury, mitochondrial permeability transition	GPX4 depletion, ACSL4 activation, labile iron accumulation, lipid peroxide formation	Respiratory chain leakage, mitochondrial swelling, membrane potential collapse	Early allograft dysfunction, primary nonfunction, enzyme release
Cholangiocytes (Lou et al., 2026)	Ischemic cholangiopathy, bile acid toxicity, peribiliary vascular injury	Oxidative membrane vulnerability and lipid peroxidation sensitivity	ROS-mediated epithelial injury and impaired energy recovery	Biliary strictures, ischemic cholangiopathy
Kupffer cells (Li et al., 2022)	Innate immune activation, cytokine release, sterile inflammation	Iron handling, ferritin turnover, inflammatory amplification of lipid oxidation	ROS-dependent cytokine production and inflammasome activation	Cytokine surge, graft inflammation, immune activation
Liver sinusoidal endothelial cells (Yinzhi et al., 2024)	Glycocalyx disruption, endothelial swelling, leukocyte adhesion, nitric oxide imbalance	Oxidative membrane injury and susceptibility to lipid peroxidation	ROS-mediated endothelial dysfunction and impaired vasoregulation	Sinusoidal congestion, hypoperfusion, microvascular failure
Neutrophils (She et al., 2026)	Reperfusion recruitment, respiratory burst, protease release	Secondary lipid oxidation through inflammatory oxidants	NADPH oxidase-derived ROS and interaction with mitochondrial injury	Reperfusion amplification, endothelial damage
Platelets (Liang et al., 2022)	Sinusoidal adhesion, thromboinflammation, microvascular obstruction	Indirect promotion of oxidative lipid injury through vascular stasis	ROS-linked endothelial interaction and inflammatory signaling	Microcirculatory failure, graft congestion
Hepatic stellate cells (Peng et al., 2022)	Stress activation, extracellular matrix remodeling, fibrogenic signaling	Sensitivity to oxidative lipid mediators	ROS-mediated activation and profibrotic transition	Chronic graft remodeling and fibrosis risk

Moving from describing hepatic ischemia-reperfusion injury to attributing injury pathways is required for a much more serious understanding of the process. The central issue is no longer if oxidative stress and inflammation occur (because they clearly do) but what oxidative and inflammatory pathways contribute to clinically relevant graft dysfunction; what biomarkers are most reliable at identifying those pathways; and how many different perioperative interventions can mitigate those pathways without negatively impacting recovery. It is that distinction that has to be made in order to evaluate anesthetic preconditioning. If it is shown that anesthetics reduce aminotransferase levels or histologic damage, then that data is useful but insufficient. In order to provide evidence of a mechanism by which a treatment works, a study would have to demonstrate that the treatment preserves mitochondrial function (mitochondrial respiratory function), limits mitochondria-generated reactive oxygen species, inhibits iron-dependent lipid peroxidation, protects sinusoidal endothelial cell function, decreases ferroptosis-specific signaling, or improves a validated outcome after transplantation.

## Ferroptosis in liver graft injury

Ferroptosis is now one of the most significant new mechanisms to interpret liver graft injury because it represents a more precise description of oxidative membrane injury, previously known and identified but poorly understood. Ferroptosis differs from apoptosis (defined by caspase activation and ordered cellular destruction) and necrosis (representative of catastrophic membrane disruption) (Dixon and Olzmann, 2024). Instead, ferroptosis occurs through iron dependent lipid peroxidation and disintegration of antioxidant defense systems (Tang and Kroemer, 2020). The number of liver transplants is increasing and becoming more mature. Although the field’s work is promising, there will need to be cautionary interpretations of the data. Hepatic ischemia-reperfusion studies are increasing in frequency citing ferroptosis, but each increase in lipid peroxidation or decline in antioxidant capacity will not definitively establish ferroptotic cell death.

Mechanistically, ferroptosis occurs when the equilibrium between the generation and detoxification of lipid peroxides is disrupted (Endale et al., 2023). The GPX4 pathway plays a central role in this process because GPX4 converts phospholipid hydroperoxides to nontoxic lipid alcohols (Nishida Xavier da Silva et al., 2022). When either glutathione levels diminish or GPX4 activity is decreased, phospholipids are converted to oxidized phospholipids (Xie et al., 2023). Continued production of oxidized phospholipids leads to loss of membrane integrity and regulated cell death. SLC7A11 enables glutathione synthesis through importation of cysteine via System Xc-, and suppression of SLC7A11 limits cystine importation, thus reducing glutathione levels, diminishing GPX4 activity, and making cells more susceptible to lipid peroxidation (Chen et al., 2025). The System Xc-/GSH/GPX4 axis is generally accepted as a central pathway for regulating ferroptosis (Wang B. et al., 2023).

In liver graft injury the GPX4-SLC7A11 axis is crucial. Both ischemia and reperfusion have been shown to produce simultaneous increases in oxidative injury and reductions in antioxidant capacity in rodent models of warm hepatic ischemia-reperfusion and in human liver transplant biopsy samples (Kojima et al., 2024; Zhao et al., 2025). Cold ischemia produces no full prevention of ATP depletion, ionic imbalance, mitochondrial dysfunction, and redox instability. Upon initiation of reperfusion, oxygen availability increases significantly; however, antioxidant systems may already be exhausted or dysfunctional (Dery et al., 2025). These conditions favor oxidative phospholipid injury. Under these conditions, a decrease in GPX4 or SLC7A11 expression may reflect not merely cellular stress but loss of a protective system capable of preventing accumulation of lipid peroxides (Zhou et al., 2022). Reductions in GPX4 may represent responses to various types of oxidative injuries. Further evidence regarding iron dependence, lipid ROS accumulation, and rescue using ferroptosis inhibitors are required to conclude that injury was caused by ferroptosis.

Iron metabolism is a second key component of ferroptosis. Free or loosely-bound iron participates in the formation of lipid peroxides through Fenton chemistry and similar radical-generating reactions (Gutteridge and Halliwell, 2024). Transplantation presents unique opportunities for studying the relationship between iron metabolism and ferroptosis because hepatocytes, Kupffer cells, and sinusoidal cells are involved in iron sequestration, recycling, and inflammatory response to iron. Ischemia-reperfusion may generate labile iron pools through degradation of ferritin, hemoxygenase activity, mitochondrial damage, and pro inflammatory signaling (Bardallo et al., 2022). Furthermore, grafts experiencing prolonged ischemia or donor conditions involving oxidative stress, hemolysis, steatosis, or systemic inflammation are at greater risk for developing lipid radicals and accelerating membrane damage (Hirao et al., 2025). Unfortunately, clinical literature remains lacking in regard to systematic evaluation of labile iron or compartment-specific redistribution of iron in liver transplant grafts. Studies frequently present gross measures of total iron staining or alterations in ferritin levels; however, these measures fail to accurately assess the redox active iron species responsible for driving ferroptosis.

Polyunsaturated fatty acid containing phospholipids are highly susceptible to oxidative injury (Mortensen et al., 2023). ACSL4 contributes to ferroptosis by accumulating polyunsaturated fatty acid chains into membranes that can be subsequently oxidized (Ding et al., 2023). LPCAT3 then incorporates these fatty acids into phospholipids (Wang et al., 2024). Accumulation of lipid peroxides ultimately disrupts cellular membranes leading to regulated cell death (Zheng et al., 2024). Malondialdehyde (MDA), 4-hydroxynonenal (4-HNE), among others serve as indicators of lipid oxidative damage; however, their presence alone cannot confirm the occurrence of ferroptosis (Zhang et al., 2025). Rather their value increases when measured in conjunction with GPX4, SLC7A11, ACSL4, lipid ROS, available iron sources, and pharmacologic rescues. A complete and accurate claim for ferroptosis should ideally demonstrate several key components collectively: increased redox-active iron; accumulation of oxidized phospholipids, impaired GPX4 or glutathione defense; increased ACSL4-associated lipid susceptibility, morphological or molecular differentiation from apoptosis and necroptosis where applicable, rescue using targeted ferroptosis interventions such as iron chelating compounds, lipid radical trapping antioxidants or pathway-specific GPX4 reconstitution therapies.

Differentiation between ferroptosis and mitochondrial injury is additionally very important since these mechanisms are both interrelated but distinct. Mitochondrial injury can lead to ferroptosis through generation of ROS; alteration of energy homeostasis; modification of iron sulfur clusters; and promotion of lipid oxidation (Bucarey et al., 2026). Concomitantly, ferroptotic membrane damage can exacerbate mitochondrial instability/inflammation (Hirao et al., 2025). Therefore studies demonstrating mitochondrial swelling, loss of membrane potential or increased mitochondrial ROS should not be presumed to represent ferroptosis until lipid peroxidation and iron-dependent cell death are documented. On the converse side studies evaluating ferroptosis without considering mitochondrial dysfunction may overlook a critical upstream stimulus to liver graft injury.

Finally, ferroptosis may have special significance in grafts obtained from donors exhibiting steatohepatitis (Li et al., 2025). Hepatocytes undergoing steatosis possess an increased substrate availability for lipid oxidation, exhibit decreased tolerance to cold preservation temperatures, display impaired mitochondrial function and exhibit increased susceptibility to disturbances of the microcirculation (Lu et al., 2021). Consequently these characteristics create a biological environment wherein lipid peroxidation and ferroptosis may dominate over injury processes occurring in nonsteatotic grafts. Clinically this is significant because steatotic livers are increasingly being considered for transplantation due to the shortages experienced with organ supply; however they remain at high risk for early dysfunction posttransplantation (Jadlowiec and Taner, 2016). A limitation of the current evidence base is that most experimental models utilizing young healthy animals rather than older steatotic or donation-after-circulatory-death livers underestimate the degree of ferroptosis occurring in clinically high risk grafts.

## Mitochondrial ROS and redox-metabolic failure

In recent years, research has focused on the role of mitochondrial reactive oxygen species in liver transplantation-associated ischemia-reperfusion injury. Research indicates that ROS is produced in response to ischemia, reperfusion, and subsequent reoxygenation (Li and Jackson, 2002). While the use of ROS as an indicator of oxidative stress is common, research suggests that mitochondrial ROS should be viewed as a mechanismally distinct mediator of graft injury (Shi et al., 2021).

Ischemia reduces mitochondrial oxidative phosphorylation as oxygen is no longer available as the final electron acceptor (Mao et al., 2024). Consequently, the normal equilibrium between substrate oxidation and ATP production is disrupted. Furthermore, ischemic metabolism disrupts the TCA cycle and allows for increased accumulation of certain metabolites such as succinate (Zhang and Lang, 2023). Upon restoration of oxygen upon reperfusion, the accumulated succinate can rapidly stimulate electron transfer through the electron transport chain. A significant amount of current data suggest that ischemic succinate accumulation stimulates reverse electron transport at complex I to produce a surge of mitochondrial ROS (Fukushima et al., 2024).

Mitochondrial ROS contributes to graft injury via multiple interrelated pathways. Firstly, ROS directly damage mitochondrial proteins, cardiolipin, components of the respiratory chain, mtDNA and membrane lipids (Wang B. et al., 2023). Secondly, ROS induce opening of the mitochondrial permeability transition pore (Yalamanchili et al., 2022). Persistent opening of the PT pore leads to loss of membrane potential, cessation of oxidative phosphorylation, swelling of the mitochondrial matrix and enhanced pro-death signaling (Bernardi et al., 2023). Thirdly, ROS lead to lipid peroxidation in the cytoplasm and membranes that link mitochondrial injury with ferroptosis associated injury (Lyamzaev et al., 2023). Fourthly, mitochondrial ROS lead to release of mitochondrial DNA and other mitochondrial damage-associated molecular patterns (DAMPs), which activate innate immunity leading to increased sterile inflammation (Lyu et al., 2023).

The relationship between mitochondrial ROS and calcium overload is particularly pertinent. Ischemia induced ATP depletion impairs calcium efflux and reperfusion can exacerbate calcium entry into the mitochondria and enhance mitochondrial calcium sequestration (Matuz-Mares et al., 2022). Elevated mitochondrial calcium enhances likelihood of PT pore opening and hastens respiratory chain dysfunction thereby creating a detrimental cycle (Belosludtsev et al., 2025). Calcium overload generates mitochondrial ROS; ROS generates additional membrane damage; membrane damage compromises calcium regulation. In liver transplantation this cycle may be particularly pronounced during initial stages of reperfusion when portal vein or arterial flow exposes the metabolically fragile hepatocytes/sinusoidal endothelial cells to sudden changes in oxygen delivery/shear stress/inflammatory mediators. Preservation of mitochondrial calcium handling may reduce injury regardless of direct antioxidant treatment. One of the largest drawbacks to existing literature is that mitochondrial ROS is often implied but not directly measured. Most studies utilize surrogate measures of total ROS, Malondialdehyde, SOD, glutathione, catalase, inflammatory cytokines and claim these indicate mitochondrial oxidative injury. While these surrogates are useful they are not specific to mitochondria.

The intersection of ferroptosis and mitochondrial ROS represents one of the most important areas of mechanistic overlap within this review. Mitochondrial ROS can stimulate lipid peroxidation, depletion of antioxidant defenses, establish redox environments conducive to ferroptosis (Wang Y. et al., 2023). Mitochondrial dysfunction can also disrupt iron-sulfur cluster metabolism and cellular iron homeostasis potentially enlarging the redox-active iron pool (Duan et al., 2023). On the converse side ferroptotic lipid peroxidation can injure mitochondrial membranes/worsen respiratory chain stability. Although these two processes are mechanistically distinct—mitochondrial ROS is a biochemical event while ferroptosis is a regulated cell death modality—they frequently co-exist and reinforce each other within the same pathological system, particularly in the context of hepatic ischemia-reperfusion injury (Pan et al., 2026; Song et al., 2025).

Finally, microvascular failure also magnifies oxidative-redox injury due to mitochondria. Swelling of sinusoidal endothelium, adhesion of leukocytes, aggregation of platelets and disruption of NO homeostasis impede O2 delivery following reperfusion. Therefore, even post-vascular declamping, tissue O2 delivery is heterogeneous in nature with some hepatocytes receiving supraphysiological amounts of O2 during initial reperfusion resulting in excessive ROS production while others remain relatively hypoxic thereby extending metabolic incompetence (Del Prete, 2026). Spatial heterogeneity is typically not reflected by serum ALT/AST values that reflect global hepatocellular injury but do not capture whether damage is confined to periportal, cholangiocellular, endothelial compartments, etc. Further advanced imaging/spatial transcriptomics/tissue compartmentalized analyses may be necessary to elucidate variability in mitochondrial ROS across the graft.

**FIGURE 1:**
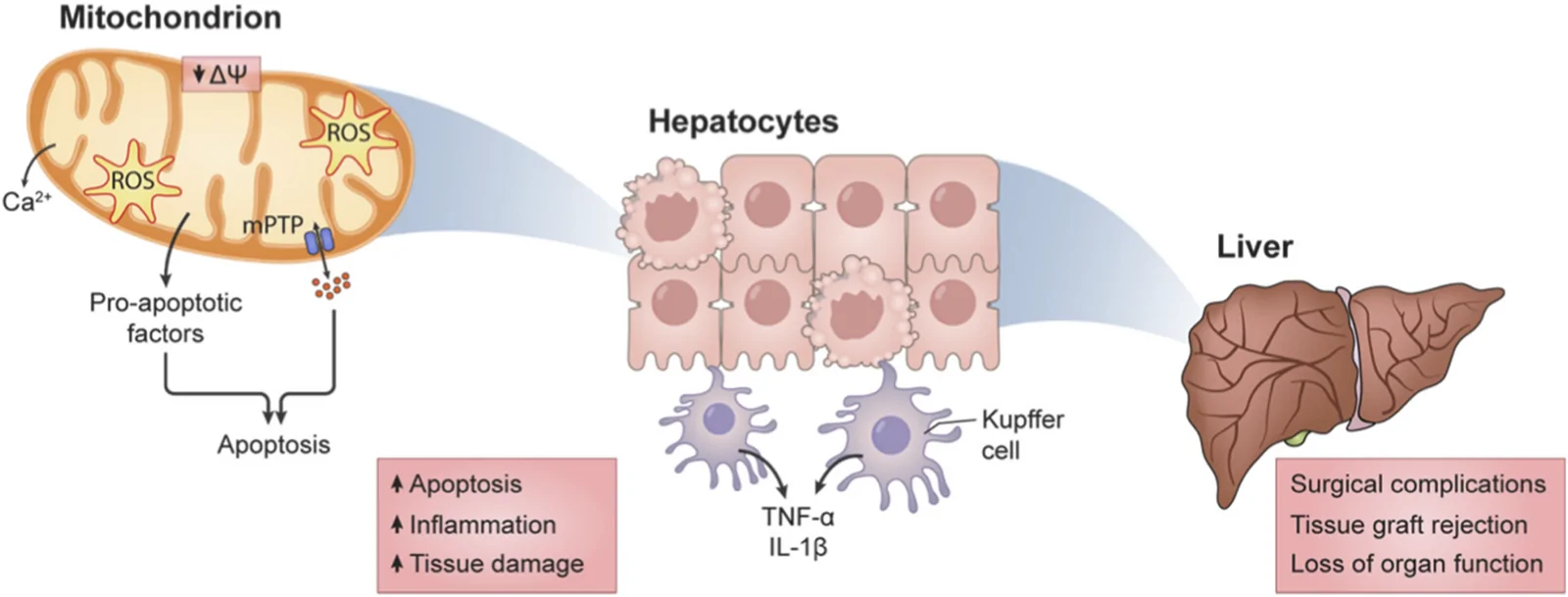
Mitochondrial ROS and mPTP opening drive cellular injury to sterile inflammation and organ failure (Teodoro et al., 2022).

Further complicating this picture is that many previous reports have indirectly implied mitochondrial involvement based on measurements indicative of oxidative stress (i.e., MDA/H2O2/O2-, SOD activity, etc.) without demonstrating its presence directly within the mitochondria. Studies evaluating ferroptosis without considering mitochondrial dysfunction—whether conducted in cell lines, animal models, or human transplant biopsies[5]—may overlook a critical upstream stimulus to liver graft injury. Figure 1 illustrates how mitochondrial failure can escalate local cellular injury into broader hepatic ischemia–reperfusion injury and, if unresolved, contribute to graft dysfunction and organ failure (Teodoro et al., 2022).

## Anesthetic conditioning as a modulator of ferroptosis and mitochondrial injury

Anesthetic preconditioning refers to the ability of certain anesthetic agents to increase tissue resistance against ischemia-reperfusion injury when used before ischemia, during ischemia or at the onset of reperfusion (Alvarez et al., 2014). This area has significant implications for liver transplant medicine where anesthesia is a routine part of managing the donor, performing recipient surgery, implanting the graft and controlling the initial reperfusion. One advantage of using anesthetic agents over other experimental organ-protective drugs is that they can be administered via a standard route (i.e., inhalational) without requiring additional routes of administration (Vaghela et al., 2023). While this makes their application feasible during clinically-critical periods; it does not confirm that they will provide benefit. The literature provides strong biological plausibility and experimental promise for using anesthetic preconditioning, however there is no consistent clinical translation due to variability in liver graft quality, time under ischemia, hemodynamic complexity and the number of overlapping injury pathways involved.

The majority of experimental data supporting anesthetic preconditioning comes from investigations involving volatile anesthetics. Studies investigating sevoflurane, isoflurane and desflurane have shown that each agent significantly reduces ischemia-reperfusion injury in experimental models by modulating mitochondrial function, redox balance, inflammatory response, and endothelial integrity. Sevoflurane has received the most attention among volatile agents because of its frequent clinical usage, ease of titration, and numerous associations with cytoprotective signaling pathways in rodent models of hepatic ischemia-reperfusion injury and in primary hepatocyte cultures exposed to hypoxia-reoxygenation (Wu et al., 2025; Zhang et al., 2022). Potential mechanisms include activating mitochondrial K(ATP) channels, activating PI3K/Akt and ERK signaling, inducing STAT3 activation, regulating HIF-1α expression, modulating nitric oxide production and stimulating NRF2 mediated antioxidant defenses (Szrama et al., 2022). Each of these pathways plays a role in protecting the liver graft from damage caused by reperfusion by maintaining mitochondrial integrity, preventing calcium dyshomeostasis, limiting ROS production and reducing inflammatory responses.

One of the primary mechanistic rationales for using volatile anesthetics to precondition tissues prior to ischemic insult is through providing protection to mitochondria. Damaged mitochondria generate large amounts of reactive oxygen species during reperfusion due to instability of the electron transport chain, impaired calcium homeostasis and the opening of the mitochondrial permeability transition pore. Volatile anesthetics could potentially limit this type of injury by preserving mitochondrial membrane potential, reducing calcium influx, inhibiting the opening of the permeability transition pore and stabilizing the function of the respiratory chain (Dalal, 2016).

At the molecular level, volatile anesthetics directly interfere with mitochondrial electron transport chain components. Harisseh et al. demonstrated that isoflurane, sevoflurane, and desflurane all inhibit complex I (NADH:ubiquinone oxidoreductase) and decrease mitochondrial membrane potential, while exhibiting divergent effects on reactive oxygen species production in isolated cardiac mitochondria (Harisseh et al., 2017). Rodriguez et al. further showed that isoflurane and sevoflurane specifically disrupt complex I-linked respiration and simultaneously increase mitochondrial membrane potential, implying reversal of the ATP synthase and consequent ATP depletion (Rodriguez et al., 2025). These actions on complex I may limit electron leakage and subsequent ROS burst upon reperfusion, contributing to the protective phenotype observed in experimental models.

Despite considerable evidence demonstrating that volatile anesthetics reduce indicators of tissue injury such as elevated aminotransferases, histological damage, malondialdehyde levels, inflammatory cytokines and oxidative stress; none of these results definitively demonstrate that these agents act through mitochondrial-specific mechanisms. To make a stronger mechanistic claim regarding mitochondrial-specific action of volatile anesthetics would require measurement of mitochondrial respiration, mitochondrial membrane potential, activities of complexes within the respiratory chain, rates of ROS production by mitochondria, rates of permeability transition pore opening and rates of recovery of mitochondrial ATP synthesis. Until such measurements are made it will remain unclear whether volatile anesthetics act directly on mitochondria or simply reduce global injury burden.

Sevoflurane represents the most supported volatile agent to utilize in anesthetic preconditioning based on the strength of mechanistic evidence available in organ-protection research. There are four mechanistically-interacting ways in which sevoflurane could exert beneficial effects. Firstly, it may prevent mitochondrial ROS production through stabilization of mitochondrial function during reperfusion (Lotz et al., 2020). Secondly, it may activate antioxidant signaling pathways to maintain glutathione metabolism and lipid peroxide detoxification (Xu et al., 2016). Thirdly, it may inhibit inflammatory-amplifying processes by limiting cytokine release, neutrophil infiltration and endothelial activation (Rancan et al., 2014). Lastly, it may improve microcirculatory stability thus reducing regional hypoxia following reperfusion (Li et al., 2016). Importantly, these four mechanism-based pathways do not interact independently. Mitochondrial ROS production, lipid peroxidation, inflammation and microvascular dysfunction are interdependent so that interventions targeting several aspects simultaneously could produce better protection than single-targeted therapies.

Isoflurane has also been demonstrated to provide ischemic tolerance (especially via mitochondrial KATP channels and survival kinases) and the protective mechanism of iso-fluorane is biologically plausable; however, liver transplantation specific data regarding iso-fluorane’s protective effects are weaker than those observed with sevoflurane (Burda et al., 2023). Desflurane has shown conditioning like protective properties and desflurane’s pharmacological characteristics make it a candidate for rapid titration, although, there exists limited understanding of how desflurane influences ferroptosis and mitochondrial ROS in liver graft injury (Djuric et al., 2025). Each agent is unique due to their differences in tissue solubility, hemodynamic effect, inflammatory influence, mitochondrial signaling, and clinical usage. Therefore, a generalization of anesthetic agents is not appropriate as each agent offers distinct mechanisms of organ protection in transplantation.

As mentioned previously, propofol presents a different paradigm of anesthetic mediated organ protection. Propofol contains a phenolic structure which lends itself to antioxidant properties (Volti et al., 2006). Additionally, propofol may inhibit lipid peroxidation thereby stabilizing mitochondrial membranes and inhibiting inflammatory signaling, as demonstrated in rodent models of hepatic ischemia-reperfusion and in cell models of hypoxia-dependent HIF activation (Ge et al., 2017; Hsing et al., 2011). Due to ferroptosis being dependent upon lipid radical propagation, propofol is particularly relevant to ferroptosis as it may reduce lipid peroxide formation and thus theoretically limit ferroptosis associated damage (Zhang et al., 2023). Furthermore, propofol may maintain mitochondrial membrane potential and lower ROS levels (Bellanti et al., 2016). However, propofol cannot be assumed to be a direct inhibitor of ferroptosis. Lipid peroxide inhibition is consistent with anti-ferroptotic activity; however, it can also result from the broader antioxidant actions of propofol. Studies demonstrating effects of propofol on GPX4, SLC7A11, ACSL4, labile iron, lipid ROS and ferroptosis sensitive rescue pathways will be required to demonstrate propofol is specifically anti-ferroptotic.

In addition to assessing anesthetic conditioning as a method for protecting the liver graft there is a growing interest in evaluating anesthetic conditioning as a tool used in conjunction with machine perfusion techniques. Machine perfusion techniques allow liver grafts to be manipulated/treated/prepared prior to implantation. With normothermic and hypothermic oxygenated machine perfusions the possibility now exists to test anesthetic-like protective strategies directly on the graft; measure perfusate biomarkers; evaluate mitochondrial function; and eliminate donor recipient confounds prior to clinical reperfusion. From a theoretical perspective, agents capable of maintaining mitochondrial integrity; reducing lipid peroxidation; activating antioxidant signaling pathways could be added into perfusates prior to clinical reperfusion. However, this area remains experimental as well as the pharmacology; tissue distribution/wash-out behaviors; biliary safety; and mitochondrial endpoint effects of such agents remain unknown until they are tested for use in humans.

Since anesthetic agents vary in terms of their physical/chemical properties; mitochondrial effects; antioxidant capacities; and immunomodulatory signaling activities, comparative analyses of their organoprotective mechanisms will require analysis across overlapping but non-identical molecular targets. Some anesthetics and their molecular mechanisms of action are as shown in the table below are shown in Table 2.

**TABLE 2:** Anesthetic agents and proposed organoprotective mechanisms.

Agent/Class	Main protective pathways	Ferroptosis-relevant effects	Mitochondrial ROS-Relevant effects	Translational strength
Sevoflurane (Li et al., 2016)	PI3K/Akt, ERK, STAT3, HIF-1α, NRF2, HO-1, mitochondrial potassium channel signaling	May preserve GPX4/SLC7A11-related antioxidant capacity, reduce lipid peroxidation, and indirectly limit iron-driven membrane injury	May reduce mitochondrial ROS burst, stabilize membrane potential, and limit permeability transition	Moderate to strong preclinical evidence; clinical translation remains variable
Isoflurane (Clarysse et al., 2023)	Mitochondrial potassium channel activation, survival kinase signaling, redox adaptation	Possible indirect reduction of lipid peroxidation through mitochondrial and antioxidant effects	May reduce reperfusion oxidative injury and preserve mitochondrial function	Moderate preclinical evidence; limited ferroptosis-specific data
Propofol (Bellanti et al., 2016)	Phenolic antioxidant activity, radical scavenging, anti-inflammatory signaling	Potential suppression of lipid radical propagation and lipid peroxidation; specificity for ferroptosis requires stronger proof	May stabilize mitochondrial membranes and reduce ROS generation	Strong antioxidant rationale; difficult to separate conditioning from general antioxidant effect

This comprehensive assessment concludes that anesthetic conditioning can provide protective effects to liver grafts (assuming the conditioning effect is successful) at the network level, as opposed to solely via a single pathway. Propofol may protect liver by reducing the generation of free radicals in lipids and antioxidants, which ultimately lead to increased activity of the NRF2 and GPX4 system (Bellanti et al., 2016). These pathways ultimately converge at the mitochondrial reactive oxygen species-ferroptosis axis but direct inhibition of ferroptosis has not been sufficiently demonstrated. Therefore, it would be more correct to say that anesthetic conditioning reduces the mitochondrial and redox conditions that allow ferroptosis to contribute to additional damage from the initial insult to the liver graft, versus stating that volatile anesthetics are clearly therapeutic against ferroptosis in liver transplantation.

Anesthetic conditioning presents a reasonable mechanism-based approach to mitigate liver graft injuries resulting from ischemia-reperfusion occurring post-transplant (Gilbo et al., 2016). Anesthetic conditioning’s greatest theoretical justification resides in its potential to affect the major factors involved in causing graft injury during the period immediately after injury occurs. Specifically, anesthetic conditioning appears capable of influencing both the generation of reactive oxygen species by mitochondria, antioxidant signal transduction pathways, inflammatory processes within the graft, endothelial function within the graft, and lipid peroxidation (Djuric et al., 2025). As such, there exists sufficient evidence to support continued exploration of this area of research, specifically regarding sevoflurane and dexmedetomidine. However, conclusive determinations regarding anesthetic conditioning as an effective protective measure in liver transplantation require pathway-specific experimental validations and randomized controlled clinical trials designed specifically around transplant scenarios. Therefore, as reviewed here, anesthetic conditioning can be viewed as a potentially useful multi-target modulator of the ferroptosis-mitochondrial ROS injury network as opposed to a fully validated anti-ferroptotic therapy.

Beyond anesthetic conditioning, the post-transplant pharmacological landscape is dominated by immunosuppressive regimens, yet their potential to modulate ferroptosis and mitochondrial ROS in liver grafts remains largely unexplored. This gap is clinically relevant because calcineurin inhibitors (tacrolimus, cyclosporine) and mTOR inhibitors (sirolimus, everolimus) are routinely administered from the reperfusion period onward, and each class exhibits distinct mitochondrial effects. Tacrolimus has been shown to induce time-dependent production of oxygen free radicals in isolated rat hepatic mitochondria, suggesting that prolonged exposure may directly contribute to mitochondrial oxidative burden in the graft (Han et al., 2006). In contrast, a recent *in vivo* study demonstrated that sirolimus (SRL) treatment over 4 weeks in mice produced a trend toward decreased hepatic cellular respiration (Albawardi et al., 2023). This inhibition of mitochondrial bioenergetics by mTOR inhibitors is consistent with the known role of mTOR in regulating mitochondrial oxidative phosphorylation. From a ferroptosis perspective, the implications are twofold. On one hand, immunosuppressants that increase mitochondrial ROS or impair ATP recovery could theoretically lower the threshold for ferroptotic membrane injury by exacerbating oxidative stress and depleting antioxidant reserves. On the other hand, agents that suppress mitochondrial respiration might reduce electron transport chain leakage and thus limit superoxide generation upon reperfusion, potentially attenuating a key upstream trigger of ferroptosis. However, direct evidence linking specific immunosuppressants to GPX4 activity, SLC7A11 expression, or lipid peroxidation in the transplant setting is currently absent. Furthermore, the interplay between anesthetic conditioning and immunosuppressive agents—whether additive, synergistic, or antagonistic with respect to ferroptosis modulation—has not been investigated. This represents a critical knowledge gap, as perioperative anesthetics and post-reperfusion immunosuppressants are administered in sequence, and their combined effects on mitochondrial redox status and ferroptotic susceptibility may determine graft outcomes, particularly in marginal livers with pre-existing metabolic compromise.

Among calcineurin inhibitors, cyclosporine warrants particular attention because its mitochondrial targets directly overlap with those of volatile anesthetics. Cyclosporine inhibits the mitochondrial permeability transition pore by binding to cyclophilin D, a mechanism that parallels the proposed action of sevoflurane and isoflurane in limiting mPTP opening during reperfusion (Huhn et al., 2008; Waldmeier et al., 2002). Importantly, volatile anesthetics target complex I of the electron transport chain, while cyclosporine has been shown to suppress both complex I- and complex II-linked respiration in skeletal muscle mitochondria (Hokanson et al., 1995). This convergence on shared mitochondrial targets raises the possibility of additive or synergistic effects when the two agents are co-administered. However, experimental evidence also suggests that the interaction is complex and context-dependent—a study in an *in vivo* rat heart model found that isoflurane, but not cyclosporine A, reduced infarct size when administered at reperfusion, leading the authors to propose that cardioprotection may require a combined effect on both cyclophilin D and complex I during the first minutes of reperfusion (De Paulis et al., 2013). This finding underscores that the interaction may depend on the timing of administration and the specific mitochondrial state. Currently, direct evidence on how cyclosporine modulates anesthetic-induced protection specifically in liver transplantation is lacking, representing a critical knowledge gap. Future mechanistic studies should investigate whether cyclosporine therapy alters the mitochondrial target of volatile anesthetics in the hepatic graft and whether this interaction impacts clinical outcomes in transplant recipients.

Future mechanistic studies should incorporate immunosuppressant exposure as a variable in experimental models of hepatic IRI and, where possible, correlate drug levels and mitochondrial function biomarkers with ferroptosis-specific endpoints in transplant recipient cohorts.

## Integrated mechanistic model, biomarkers, and therapeutic translation

The combination of the impact of ferroptosis and microvascular injury along with the increased levels of ROS, the presence of systemic inflammation, the degree of endothelial dysfunction, and subsequent microvascular failure provide a better basis to explain liver graft injury than a singular pathogenic injury. Ischemia-reperfusion injury of the liver cannot be solely attributed to either oxidative stress or to inflammation (Choi and Lim, 2023). Upon reperfusion, a self sustaining cascade occurs where the impairment of mitochondrial structure leads to an increase in ROS, leading to lipid peroxidation through the action of ROS, and lipid peroxidation leads to ferroptotic injury of cellular membranes. Once cellular injury has occurred, the dying cells release damage associated molecular patterns (DAMPs) which trigger the activation of Kupffer cells, neutrophils, complement systems, and endothelial responses (Mihm, 2018). As a result of the inflammatory response produced by these events, there will be additional worsening of sinusoidal stasis, tissue hypoxia, and secondary mitochondrial injury (Nastos et al., 2014). Thus, this is a cyclical event rather than a linear sequence of events.

The model described above can also explain why many single target therapeutic strategies have failed to achieve significant clinical results. Total elimination of ROS may not be sufficient to prevent injury when other mechanisms of injury (iron dependent lipid peroxidation, endothelial edema, and enhanced inflammatory stimulation) continue to operate. Similarly, while reducing lipid injury from ferroptosis may be beneficial for reducing lipid injury, restoration of mitochondrial ATP production and improvement in microvascular perfusion will not occur. Consequently, protective interventions in the clinic would likely need to employ multi-site intervention in order to modulate the stability of mitochondria, remove lipid radicals and toxic metabolites from the system, minimize excessive inflammatory responses, and restore sinusoidal flow.

While mitochondrial ROS plays an important role in this network due to its ability to cause injury to mitochondrial components upon reoxygenation, it should not be viewed as the sole source of injury. Mitochondrial injury caused by ischemia involves multiple factors including energy depletion, calcium overload, decrease in respiratory chain activity, and metabolic changes all of which make the liver susceptible to oxidative stress (Go et al., 2015). Upon reoxygenation, mitochondrial ROS quickly becomes elevated and causes structural damage to the respiratory enzymes, cardiolipin, mitochondrial DNA and membrane lipids. The structural damage caused by ROS to the mitochondria enhances the likelihood that there will be a transition pore opening, loss of membrane potential, impaired ATP recovery, and inflammatory signaling (Belosludtsev et al., 2025). Additionally, in organs rich in iron and capable of lipid metabolism such as the liver, mitochondrial ROS can enhance lipid peroxidation thus providing a direct link between mitochondrial dysfunction and ferroptosis (Muriel, 2017).

Ferroptosis adds a level of specific detail to our overall concept of oxidative injury. While both concepts are based on the formation of reactive species in the cell, the defining feature of ferroptosis is the rapid iron-catalyzed oxidation of phospholipids that overwhelm the anti-ferroptotic defenses provided by GPX4, glutathione, SLC7A11, etc., (Wu et al., 2021). Transplant livers are particularly vulnerable to this type of injury as hepatocytes, cholangiocytes and sinusoidal endothelial cells are at risk for high redox stress during reperfusion. Ferroptosis however should not be used interchangeably with lipid peroxidation. Evidence that lipid injury is indeed iron dependent, biologically relevant and represents regulated cell death will be required before it can be considered as a valid biological mechanism.

**FIGURE 2:**
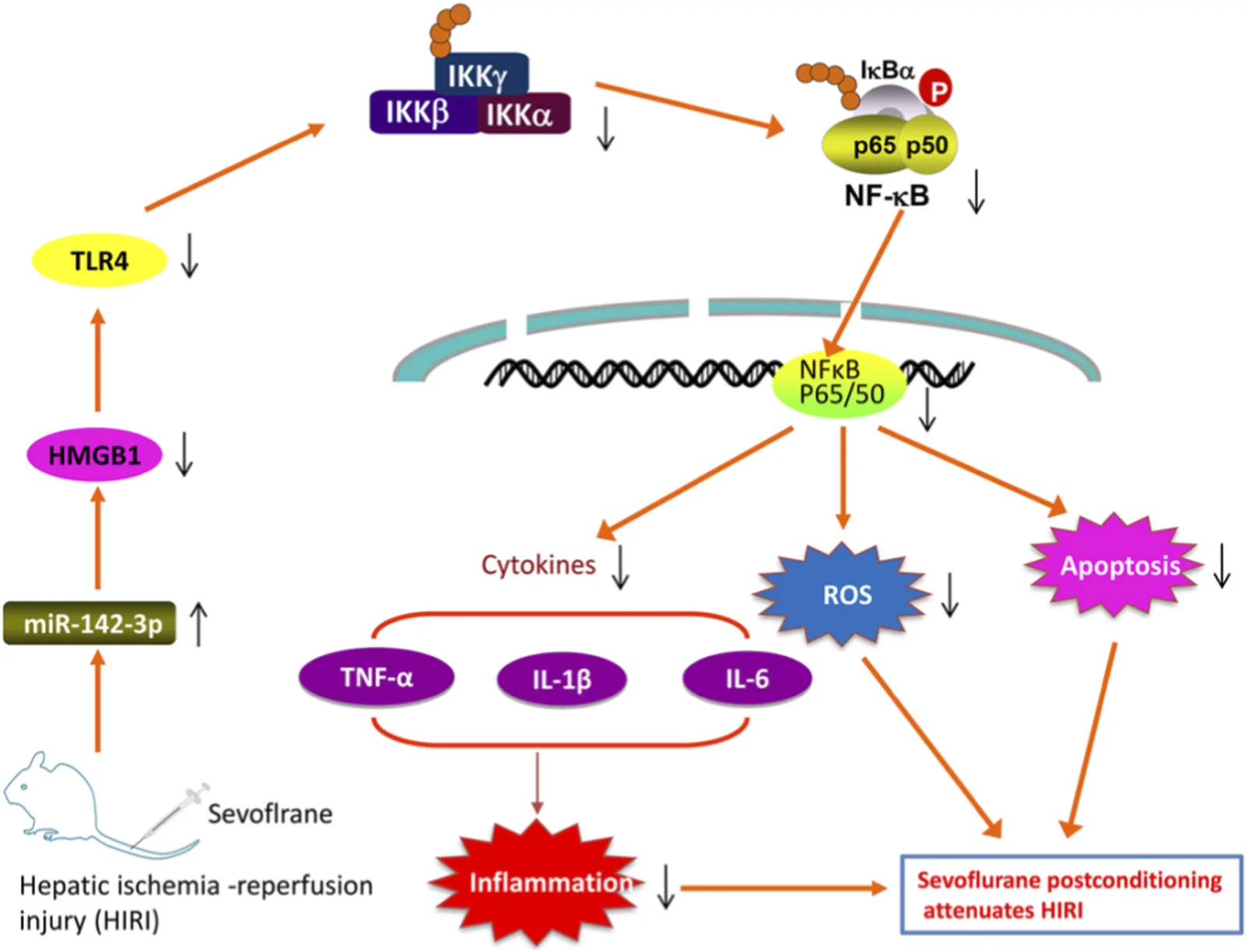
Sevoflurane postconditioning attenuates HIRI via miR-142/HMGB1/TLR4/NF-κB pathway (Xu et al., 2021).

Given that volatile anesthetics can act on multiple sites within this injury network (stabilize mitochondrial function, activate survival kinase cascades, decrease mitochondrial ROS formation, enhance antioxidant signaling), propofol can inhibit lipid radical propagation and suppress inflammatory oxidative damage; dexmedetomidine can modulate sympathetic stress/inflammatory signaling/mitochondrial dysfunction/antioxidant signaling), the key translational issue is whether these effects are strong enough and timely enough to produce measurable improvements in graft outcome. Figure 2 depicts anesthetic conditioning as an upstream modulator of mitochondrial ROS, antioxidant defense, lipid peroxidation, ferroptosis, inflammatory activation, endothelial function, and graft microcirculation, with downstream effects including reduced hepatocellular injury, improved sinusoidal perfusion, lower biliary injury risk, and improved early graft function (Xu et al., 2021).

The ability to develop biomarkers for assessing the various pathways of graft injury and disease is crucial to translate this basic science knowledge to clinical applications. The use of traditional liver dysfunction parameters (ALT/AST, bilirubin, INR, lactate, bile production, and histology) continues to have utility but lack specific relevance to the pathologic process. For example, ALT and AST are indicators that hepatocytes are injured; however, they do not provide information about the relative contribution of mechanisms (ROS mediated mitochondrial damage, ferroptosis, apoptosis, necrosis, etc.) to this injury (Tang et al., 2022). Thus, the assessment of ferroptosis should utilize multiple markers including GPX4, SLC7A11, glutathione status, ACSL4, labile iron, ferritin related indices, lipid ROS, malondialdehyde (MDA), 4-hydroxynonenal (4-HNE), and oxidized phospholipids (Chen et al., 2024). Similarly, the evaluation of mitochondrial damage, injury should incorporate measurements of mitochondrial membrane potential/respiratory chain activity, oxygen utilization, ATP recovery, mitochondrial DNA release, cardiolipin oxidation and permeability transition susceptibility (Mihajlovic and Vinken, 2022).

Machine perfusion represents a potential bridge between the identification of the underlying mechanisms of injury and therapeutic interventions (Czigany et al., 2020). Both hypothermic oxygenated perfusion and normothermic machine perfusion enable pre-transplant assessment of graft viability and potentially provide a means for targeting protective interventions directly to those grafts with evidence of functional impairment. Such assessments might include measurement of lactate clearance during perfusion, bile quality, oxygen consumption, transaminase release, donor-derived inflammatory marker levels, ferroptosis associated markers and mitochondrial injury markers to assist in identifying those grafts that require further treatment prior to transplantation. These types of approaches may facilitate the implementation of a “precision medicine” strategy whereby steatotic grafts receive enhanced anti-lipid peroxidative treatments or donation after circulatory death (DCD) grafts receive mitochondrial stabilizing therapies and/or inflamed grafts receive either anti-inflammatory or microvascular supporting interventions.

In summary, anesthetic preconditioning should be considered as one of several modifiable perioperative influences affecting a larger graft injury disease network rather than as a single pathway intervention. Therefore its clinical benefit will depend upon its ability to diminish mitochondrial derived reactive oxygen species/preserve anti-ferroptotic defense/limit lipid peroxide formation/reduce inflammation/provide protection to vascular endothelium/and improve microvascular reperfusion/recovery. Future research needs to evolve beyond comparative descriptions of different anesthetics towards mechanistic confirming trials utilizing pathway specific biomarkers/grant risk stratification/clinically relevant endpoints.

## Limitations, challenges, and future directions

As interest grows about ferroptosis, ROS generated in mitochondria and anesthetic preconditioning; current data has been limited due to methodological heterogeneity. Most contemporary studies examining liver ischemia-reperfusion injury continue to employ a wide array of general markers of injury including aminotransferases, histologic necrosis, total ROS, malondialdehyde, superoxide dismutase activity, and pro-inflammatory cytokines. The majority of these injury markers assess the degree of damage and are unable to determine if protection occurs through reduction of mitochondrial ROS generation, inhibition of ferroptosis, enhanced endothelial function, attenuation of inflammation, or some combination of pathways.

The most significant limitation is that there is no consensus regarding what constitutes ferroptosis. While various combinations of low levels of GPX4, high levels of ACSL4, elevated levels of free-iron, and lipid peroxidation may indicate ferroptosis susceptibility; none are specific to confirm ferroptotic cell death. Lipid peroxidation can result from mitochondrial injury, neutrophil activation, toxicities resulting from bile acids, necrosis, and non-specific oxidative stress.

Therefore, strong studies would need to utilize combinations of lipid ROS (e.g., MitoSOX), labile iron (e.g., Fe2+), GPX4 activity, SLC7A11 expression, ACSL4 expression, oxidized phospholipids (e.g., cardiolipin oxidation), and rescue with ferroptosis-targeted therapeutic agents (e.g., Ferrostatin).

Similar limitations exist when interpreting the impact of mitochondrial ROS production. Total ROS assays cannot differentiate among oxidants produced by mitochondria versus those produced by NADPH oxidases, xanthine oxidase, inflammatory cells, cytochrome P450 enzymes, or lipid radical propagation. Therefore future studies would need to utilize mitochondrial-specific probes, measures of respiratory chain function (e.g., citrate synthase activity) and membrane potential (e.g., JC-1 staining), assessments of mitochondrial DNA release, measures of oxygen consumption (e.g., Clark electrode), rates of ATP recovery, and assessments of permeability transition pore opening.

Although repeated clinical sampling is often challenging, assessing each organ system involved will provide greater mechanistic understanding.

In addition to the previously mentioned limitations, the translational relevance of preclinical models warrants careful consideration. While the use of healthy young animals subjected to brief, controlled ischemic periods is methodologically appropriate for isolating specific mechanistic variables and minimizing confounding factors, this approach does not fully capture the clinical complexity of human liver transplantation. In the clinical setting, graft injury is shaped by multiple interacting factors—including donor age, hepatic steatosis, donation-after-circulatory-death status, cold and warm ischemia times, hemodynamic instability, vasopressor support, immunosuppressive therapy, and recipient comorbidity burden—that are difficult to recapitulate in a single animal model. Consequently, findings from reductionist experimental systems may not directly translate to the heterogeneous patient populations encountered in transplantation practice.

Anesthetic preconditioning is also highly variable in clinical practice as the ability to demonstrate protective effects depends upon the type of agent administered, the dose administered, the time at which it was administered relative to the onset of ischemia, the duration over which it was administered, the partial pressure of oxygen administered concurrently with anesthesia, and/or concurrent administration of other drugs.

Therefore future studies should incorporate more phenotype-based study designs. For example, grafts harvested from livers containing macrosteatosis may require targeted anti-lipid peroxidative strategies; while grafts procured using a donation after circulatory death strategy may require protective interventions designed to preserve mitochondrial and biliary microvascular integrity. Grafts procured after long durations of ischemia may require additional therapies directed towards inhibiting succinate-driven ROS production and endothelial activation.

Biomarker panels incorporating both ferroptosis markers (e.g., lipid ROS and labile iron) and functional measures of mitochondrial health (e.g., ATP recovery rate and membrane potential) along with samples collected from graft tissue, blood and bile as well as machine perfusion fluid could provide insight into the molecular events underlying graft injury in humans.

Machine perfusion may offer particular advantages as it permits for real-time assessment of graft viability and simultaneous delivery of protective interventions prior to surgical implantation.

Ultimately, the problem is not the presence of a plethora of biologically plausible mechanisms to explain why preconditioning protects against liver ischemia-reperfusion injury. Rather, the difficulty lies within a lack of standardization and transplant-specific validation. Therefore future trials should include risk stratification based on graft characteristics; detailed reports concerning exposure to anesthetics; biomarkers capable of distinguishing among different pathways (e.g., ferroptosis markers); and clinically relevant outcome measures such as early allograft dysfunction; ischemic cholangiopathy; ICU admission and duration; graft survival; and overall patient survival.

## Conclusion

The mechanisms underlying liver transplantation associated with ischemia-reperfusion injury are well-defined by the inter-related biochemical events of; metabolic arrest, oxygen restored, calcium overload, mitochondrial instability, iron mobilized, lipid peroxidation, inflammatory response and microvascular failure. The biochemical events identified above have been shown to occur in concert. For example, mitochondrial ROS has been shown to increase lipid peroxidation and ferroptosis. In addition, ferroptotic membrane damage has been demonstrated to enhance inflammation, endothelial dysfunction and graft hypoperfusion. The association of these injury networks are particularly relevant in livers obtained from marginal donors (steatotic, DCD, P-I) where there exists an increased risk of poor post-transplant function.

A new area of research that could potentially modify multiple aspects of the injury networks outlined above is anesthetic conditioning. Volatile anesthetics have been reported to stabilize mitochondria and initiate adaptive cellular survival signals. Propofol has been documented to inhibit lipid free radical formation and subsequent oxidative injury. Dexmedetomidine may modulate both inflammatory responses and sympathetic stress as well as mitochondrial dysfunction and antioxidant pathways. Currently however, anesthetic conditioning cannot be described as an anti-ferroptotic treatment due to lack of strong mechanistic data validating its use in preventing ferroptotic injury in liver transplant patients. Anesthetic conditioning appears to mitigate the mitochondrial and redox environment that allows ferroptosis and inflammatory injury to develop.

Further studies evaluating the potential benefits of anesthetic conditioning must include additional mechanistic validation including evaluation of specific ferroptosis panel markers, measurement of mitochondrial function, perfusate bio-markers, bile bio-markers and transplant related outcome measurements. In addition, using graft-risk stratified models combined with machine perfusion systems may provide greater specificity to identify donor livers that would be at greatest risk of benefiting from anesthetic conditioning strategies. Thus, the clinical utility of anesthetic conditioning will ultimately depend upon demonstration of consistent reduction of mitochondrial ROS levels, preservation of anti-ferroptotic defense mechanisms and improvement in endothelial/biliary compartmental viability and overall graft function in patients undergoing liver transplantation.
